# Analysis of health infrastructure and suicide rates in Brazil: a nationwide ecological spatial–temporal study, 2009–2023

**DOI:** 10.1016/j.lana.2026.101519

**Published:** 2026-06-03

**Authors:** Caibe Alves Pereira, Juliana Minardi Nascimento, Mateus Grellert da Silva, Jônata Tyska Carvalho, Manuella Pinto Kaster

**Affiliations:** a Laboratory of Translational Neurosciences, Department of Biochemistry, Federal University of Santa Catarina (UFSC), Florianopolis, Santa Catarina, Brazil; b Institute of Informatics, Federal University of Rio Grande do Sul (UFRGS), Porto Alegre, Rio Grande do Sul, Brazil; c Informatics and Statistics Department, Federal University of Santa Catarina (UFSC), Florianópolis, Brazil

**Keywords:** Suicide, Brazilian Unified Health System, Health infrastructure, Psychosocial care centers, Machine learning

## Abstract

**Background:**

Suicide remains a major global health concern and disproportionately affects low- and middle-income countries (LMICs), where socio-economic stressors and limited healthcare resources contribute to higher burdens. This study characterizes annual patterns of suicide rates in Brazil from January 2009 until December 2023, across its five geographic regions (North, Northeast, Southeast, South, and Central-West). Additionally, we constructed a harmonized dataset integrating sociodemographic and healthcare infrastructure indicators, and identified which municipal-level infrastructure components are most informative for classifying suicide rate categories.

**Methods:**

We integrated nationwide data from the Mortality Information System (SIM/SUS), the National Registry of Healthcare Establishments (CNES), and demographic estimates from the Brazilian Institute of Geography and Statistics (IBGE). Primary analyses were conducted using municipality-level annual observations across all Brazilian cities covering more than 170,000 suicide deaths nationally. Suicide was expressed annually as rates per 100,000 inhabitants and categorized as low, moderate, or high. Using a data-mining workflow with XGBoost classifier models, we identified healthcare infrastructure features most relevant for distinguishing suicide rate categories. Secondary analyses focused on the sociodemographic characterization of suicide deaths and on the distribution of Psychosocial Care Centers (CAPS), as well as their correlation with suicide rates.

**Findings:**

Across the descriptive analyses, suicide rates increased in all Brazilian regions (2009: 4·5, 95% CI 4·4–4·6; 2023: 7·6, 95% CI 7·5–7·7). Machine learning models identified healthcare infrastructure components (e.g., healthcare establishments, registered professionals, primary care units, diagnostic services, and mental health facilities) as the most informative features for distinguishing municipalities by suicide rate categories (low, moderate, or high), with accuracy across regions ranging from 72% (95% CI 69–75%) to 79% (95% CI 76–81%). CAPS availability was correlated with lower suicide rates across all regions (ρ = −0·71 to −0·81, p < 0·01).

**Interpretation:**

In this exploratory analysis, general healthcare infrastructure features are associated with variations in suicide mortality, suggesting that access to basic and mental health services is an informative predictor for distinguishing suicide rate patterns across regions. Findings at the aggregate level cannot be assumed to be consistent at the individual level.

**Funding:**

Santa Catarina State Research and Innovation Support Foundation (FAPESC) and the Research Program for the Unified Health System (PPSUS); Brazilian National Council for Scientific and Technological Development (CNPq); Department of Science and Technology of the Secretariat of Science, Technology, Innovation and the Health Economic-Industrial Complex of the Ministry of Health of Brazil (Decit/SECTICS); and Coordination for the Improvement of Higher Education Personnel (CAPES).

**Research in context**

**Evidence before this study**

We searched PubMed using combinations of the terms “suicide,” “healthcare infrastructure,” “Brazil,” “machine learning,” “psychosocial care centers,” and “suicide prevention.” Most previous studies in Brazil have focused on descriptive analyses of suicide trends or on the sociodemographic and clinical profiles of individuals at risk. Machine learning studies have largely targeted individual-level predictions rather than population-level outcomes. Research examining healthcare infrastructure is limited, and the role of psychosocial care centers (CAPS) in suicide prevention remains underexplored.

**Added value of this study**

This study applied data mining and machine learning to analyze suicide deaths in Brazil in the context of healthcare infrastructure across the country's five regions. Using 15 years of data (January 2009–December 2023), we assessed the influence of general healthcare services, SUS establishments, healthcare professionals, and CAPS on regional suicide rates. Our approach allowed us to identify modifiable factors associated with suicide, demonstrate the correlations of CAPS, and reveal both regional differences and commonalities in factors associated with suicide mortality.

**Implications of all the available evidence**

Our findings highlight the critical role of healthcare, including primary care, specialized services (especially public services and professionals), and CAPS, in associating key components with suicide rates. These results can inform the design of targeted public health strategies, school- and community-based prevention programs, and resource allocation policies. Strengthening and expanding mental health services, integrating culturally sensitive approaches, and addressing social determinants could reduce suicide rates not only in Brazil but also in other Latin American countries facing similar challenges.

## Introduction

Suicide is a leading global cause of death, accounting for approximately 700,000 fatalities annually. The global suicide rate in 2019 was estimated at 9 per 100,000 individuals and varied significantly across regions and countries.^1^ The vast majority of suicide-related deaths occur in low- and middle-income countries (LMICs), where patterns of suicide differ from those in high-income settings, including a disproportionately high incidence among adolescents.^2^ In these contexts, socio-economic risk factors such as poverty, unemployment, indebtedness, and unequal access to social, healthcare, and protection services among different demographic groups constitute critical risk factors.^3^^,^^4^

While global suicide rates decreased between 2000 and 2021, the Americas stood out as the only region to experience an increase, with a 17% rise over this period.^3^ In Latin America, nearly 100,000 people die by suicide each year, with the rate reaching close to 10 per 100,000 inhabitants in 2021.^3^ Within this regional landscape, Brazil represents a particularly concerning case, with suicide rates rising by 43% from 2000 to 2019.^5^^,^^6^ As the largest and most ethnically diverse population in Latin America, Brazil also displays marked regional heterogeneity in ethnic composition, economic conditions, urbanization, and, importantly, access to healthcare infrastructure.^7^ These disparities make Brazil a critical setting for understanding how variations in health system organization and service availability influence suicide risk and outcomes.

Suicide rates can often be reduced through timely public health measures and effective mental health interventions. Although preventing an individual death by suicide may rely on interventions delivered within the mental health sector, there is growing recognition that successful approaches require a broader public health perspective.^8^ While social determinants play a central role in shaping suicide risk, they have historically received limited attention in policy development and planning. Addressing these challenges requires a multifaceted approach that considers the specific risk factors associated with suicidal behavior in each region.^9^

Despite the large number of analyses of suicide rates at the global, national, and regional levels, studies that explore the characteristics of suicide, especially in LMICs, are relatively scarce. In Latin America, this scarcity of evidence is particularly concerning, given the rising burden of suicide and the unique risk profiles of its populations. A major barrier is the limited availability of reliable data, hindered by underreporting, misclassification of deaths, fragile surveillance systems for suicide and suicide attempts, and cultural stigma surrounding mental health.^1^ Strengthening data collection and surveillance systems is therefore essential to guide prevention strategies that are evidence-based and context-sensitive.

At the population level, different factors can impact suicide behavior, including social stressors (e.g., fear of crime and harassment), city infrastructure, noise and pollution, along with the quality of the health system and support.^10^ Although approximately 80% of individuals who die by suicide seek healthcare in the year preceding their death, most encounters occur in general medical settings and do not include mental health evaluations, representing a missed opportunity for prevention.^11^, ^12^, ^13^

Health infrastructure encompasses all components necessary to deliver healthcare services, ranging from disease prevention and health promotion to treatment and emergency response.^14^^,^^15^ In the context of suicide prevention, this infrastructure usually focuses on mental health services.^14^ However, in LMICs, suicide behavior does not always correlate solely with psychiatric disorders, highlighting the need to consider broader social, economic, and environmental determinants.^4^^,^^16^ Therefore, a comprehensive public health approach, targeting the entire health system and promoting universal access to preventive, promotive, and responsive care, has the potential to exert a broader and more sustainable impact on suicide prevention across diverse populations in Latin America.^17^ Most studies applying machine learning to suicidal behavior have focused on clinical prediction, emphasizing individual symptoms, behaviors, and personal characteristics to estimate the risk of suicide attempts or death.^18^^,^^19^

The primary aim of this study was to investigate whether municipal-level healthcare infrastructure is associated with variation in suicide mortality across Brazil between 2009 and 2023. We specifically tested whether structural healthcare indicators discriminate municipalities classified as having low, moderate, or high suicide rates. The secondary aims were to (*i*) characterize regional patterns of suicide mortality across Brazil's five geographic regions and (*ii*) examine the distribution of Psychosocial Care Centers (CAPS), the cornerstone of Brazil's community-based public mental health system, and their association with suicide rate categories. This systems-level approach is particularly relevant for addressing mental health challenges in low- and middle-income countries, where structural constraints and social inequalities shape access to care.^20^

## Methods

### Study design and analytical framework

This nationwide ecological spatial–temporal study analyzed municipality-level annual data from Brazil between January 2009 and December 2023. The unit of analysis was the municipality–year. The study comprised one primary analysis examining the association between healthcare infrastructure and suicide rate categories, and two secondary analyses: a descriptive epidemiological analysis of suicide mortality and a focused analysis of Psychosocial Care Centers (CAPS).

### Primary objective: healthcare infrastructure and suicide rates

Our primary research question was whether municipal-level healthcare infrastructure indicators were associated with variation in suicide mortality rate across Brazil. Specifically, we evaluated whether annual healthcare infrastructure variables discriminated municipalities classified as having low, moderate, or high suicide rates. Suicide rates were categorized according to the WHO criteria as low (≤5), moderate (>5–10), or high (>10 per 100,000). Predictors (Supplementary Table S1) were annual municipality-level healthcare infrastructure indicators.

### Data sources and linkage

The registry of suicide deaths in Brazil is compulsory and classified according to the International Classification of Diseases, 10th Revision (ICD-10). Although data are publicly available through the Unified Health System platform (DATASUS), all personal identifiers are removed before release. The system provides anonymized records, including information on circumstances of death, municipality of occurrence, and selected sociodemographic variables, without any data that could directly identify individuals.^21^

DATASUS also stores healthcare infrastructure data in the National Registry of Healthcare Establishments (CNES). The CNES database includes various categories of healthcare infrastructure, updated monthly, such as the number of hospital beds, types of healthcare facilities, availability of medical equipment, specialized services provided, healthcare teams, professional qualifications, and workforce distribution, distinguishing between public and private institutions. The healthcare infrastructure was used as predictors.

Population estimates (whole population, sex and age groups) were obtained from the Brazilian Institute of Geography and Statistics (IBGE)^7^ annually for each region throughout the study period. Race/ethnicity were self-reported and classified according to the IBGE categories as White (Branca), Black (Preta), Mixed (Parda), Asian (Amarela), or Indigenous (Indígena). Proportions were estimated through linear interpolation between the data from the 2010 and 2022 censuses, and marital status data were derived from the 2010 census, as IBGE does not produce yearly estimates for these demographic variables.

**Fig. 1:**
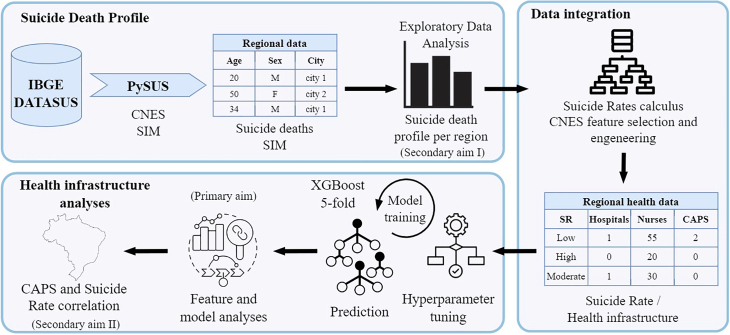
**Workflow from data collection and integration to analysis.** We collected suicide and healthcare infrastructure from DATASUS using the PySUS library, and population data from IBGE manually. Then, we constructed the first dataset using the characteristics of individuals to profile suicide death in the regions. Then, we calculated SR per city per year, integrating with the healthcare infrastructure in a new dataset. This dataset was used to predict SR. We performed feature and model analyses and observed that CAPS was relevant to the models, thus, we focused the analysis on SR and CAPS. IBGE, Brazilian Institute of Geography and Statistics; DATASUS, Unified Health System platform; CNES, National Registry of Healthcare Establishments; SIM, Mortality Information System; SR, suicide rate; CAPS, Psychosocial Care Center.

The datasets and the workflow used to modify them, including data dictionaries, were constructed by integrating data from IBGE, SIM, and CNES in Python and are available in the following repository: https://github.com/caibealves/BrazilianHealthcareDatasets/. The workflow, from data collection through analysis, is presented in Fig. 1.

### Data pre-processing and variable construction

Curated data from the SIM and CNES datasets were retrieved from the DATASUS database. Download from DATASUS was facilitated using the PySUS package in Python, a tool specifically designed to manage DATASUS data and support data analysis.^22^ To profile the populations who died by suicide in the different regions, we downloaded the death registries present in the SIM dataset. The dataset was segmented by the five Brazilian regions and filtered to include only deaths classified as suicide (we collected the whole population of suicide deaths), excluding accidents, homicides, and other causes. Additionally, observations were restricted to individuals aged 10 years and older, considering the rarity of suicide in this population and that the correct identification of suicide in young children presents significant challenges.^23^^,^^24^

The initial dataset contained 96 variables, from which we selected nine to characterize the population: age, sex, date of death, race/ethnicity, schooling, place of death, municipality, state, and ICD-10 underlying cause of death. Age was grouped into 10-year intervals, and the date of death was split into year and month. Missing data for race/ethnicity, marital status, and schooling were recorded as “ignored”, consistent with the options available in the SIM form. These missing values typically occur when the SIM form is not fully completed, and the information is absent from the deceased's records. Missing values for age or date of death were rare (<1%) and were excluded. Because this dataset is used for all causes of death nationally, many variables refer to contexts unrelated to suicide, such as chronic disease management or hospitalization details. We normalized the variables for sex, age, race/ethnicity, and marital status by the population size of the corresponding variable in each region and adjusted for the number of years. The resulting data for these variables are presented as the number of suicide deaths per 100,000 inhabitants. Schooling and cause of death are presented as the percentages of each class per the total deaths.

In the CNES dataset, we utilized eight descriptors: association with SUS; number of available hospital beds; professional categories (17 values); types of health establishments (35); healthcare team categories (7); available equipment (9); healthcare team qualifications (29); and specialized services offered (39) (Supplementary Table). We performed one-hot encoding to expand each value into a new descriptor and aggregated these values by city code and year (e.g., code 123, year 2012). Those descriptors were used by the machine learning models to classify the suicide rate of the city by year. Not all cities had all descriptors; thus, missing values (i.e., values that did not exist) were set to zero before aggregation.

For the machine learning classification, we calculated the suicide rate for each city by year. We integrated the dataset with an IBGE dataset containing the population size of each city, normalized to 100,000 inhabitants, and added a column classifying the suicide rate based on WHO recommendations: low suicide rate (SR ≤ 5), moderate suicide rate (5 < SR ≤ 10), and high suicide rate (SR > 10).^9^ Exposures consisted of annual city-level counts of healthcare establishments, workforce categories, services, and equipment, obtained from CNES and aggregated by municipality and year.

### Machine learning analysis

A pilot study was first conducted using data from the Southern region of Brazil to evaluate the suitability of different machine learning algorithms for classifying municipal suicide rates. This initial analysis indicated that the Extreme Gradient Boosting (XGBoost) algorithm, a supervised method based on gradient-boosted decision trees, was the most effective predictor.^25^ In XGBoost, each successive tree is trained to correct the residual errors of the preceding trees. The model optimizes regularization and training loss through an additive boosting framework, iteratively improving performance by minimizing prediction errors.^26^

The outcome of the machine learning models was the suicide rate. Five independent models were trained, one for each Brazilian region (North, Northeast, Central-West, Southeast, and South), using annual data at the city level. The predictor variables represented indicators of healthcare infrastructure, including the number of hospitals, physicians, nurses, and other healthcare resources available in each city and year. To ensure robust generalization and to prevent data leakage, we implemented a nested cross-validation strategy. In the outer loop, cities were partitioned into five non-overlapping folds such that each model was trained on a subset of cities and tested on unseen cities. This design guarantees that no city appeared simultaneously in training and testing sets, thus evaluating the model's ability to generalize to new geographic contexts. Within each outer training fold, an inner 3-fold cross-validation was used for hyperparameter optimization of XGBoost.

Model performance was assessed on the outer test folds using four metrics: accuracy, precision, sensitivity, and F1-score (mean and standard deviation of the five folds, with a 95% confidence interval (CI) calculated using bootstrap resampling with 1000 iterations). Additionally, multiclass areas under the receiving operating characteristic (AUROC) curves were plotted to visualize the average discriminative performance across classes. Feature importance, based on Gain, which quantifies the impact of each feature on improving the model's objective function, was extracted from each trained model to identify the most influential healthcare indicators. The ten most important features were retained for interpretation and further analysis. To explore their relationship with the outcome, Spearman correlation coefficients were computed between each top feature and the suicide rate.

### Secondary objective 1: descriptive analysis of suicide mortality

Our secondary aim was an exploratory analysis of suicide rates and the profile of people who died by suicide in the five Brazilian regions. Suicide deaths were calculated using cases classified as intentional self-harm (ICD-10) from SIM and expressed as the annual municipal suicide rate per 100,000 inhabitants, with 95% confidence intervals estimated assuming a Poisson distribution of death counts. For the initial descriptive analysis, annual suicide rates per 100,000 inhabitants were calculated for each municipality and aggregated across Brazil's five geographical macroregions. Sociodemographic characteristics (sex, age group, race/ethnicity, marital status, schooling) and methods of death were summarized as rates per 100,000 inhabitants or proportions, as appropriate.

Accumulated suicide rates were calculated as the total number of deaths in each demographic category divided by the corresponding person-years of exposure, multiplied by 100,000. The 95% CI for suicide rates were calculated assuming a Poisson distribution of deaths. The standard error of the rate was estimated as the square root of the number of deaths divided by the corresponding person-years of exposure, expressed per 100,000 inhabitants. For proportions presented in Table 1 (schooling and cause of death), the 95% CI were calculated using binomial distributions.

**Table 1:** Cumulative rates of sociodemographic characteristics of suicide deaths in Brazil from January 2009 to December 2023.

	North	Northeast	Central-West	Southeast	South	Brazil
Number of deaths	12,706	40,366	16,179	64,319	40,468	174,038
Accumulated rate	4·8 (4·7–4·9)	4·8 (4·8–4·9)	6·9 (6·8–7·0)	5·0 (4·9–5·0)	9·2 (9·1–9·2)	5·7 (5·6–5·7)
Sex						
Females	2·1 (2·0–2·2)	1·9 (1·9–1·9)	3·0 (2·9–3·1)	2·1 (2·1–2·2)	3·7 (3·6–3·8)	2·4 (2·3–2·4)
Males	7·5 (7·4–7·7)	7·9 (7·8–8·0)	10·8 (10·6–11·0)	7·9 (7·9–8·0)	14·9 (14·7–15·0)	9·1 (9·1–9·2)
M/F ratio	3·6	3·8	3·4	3·4	3·8	3·6
Age groups						
10–19	4·5 (4·3–4·7)	2·3 (2·3–2·4)	4·6 (4·4–4·9)	2·0 (2·0–2·1)	3·7 (3·5–3·8)	2·8 (2·7–2·8)
20–29	8·1 (7·8–8·4)	6·0 (5·9–6·2)	9·5 (9·2–9·8)	6·3 (6·2–6·4)	9·5 (9·3–9·8)	7·1 (7·0–7·2)
30–39	6·5 (6·2–6·7)	6·2 (6·1–6·3)	8·7 (8·4–9·0)	6·9 (6·8–7·0)	10·4 (10·1–10·6)	7·3 (7·2–7·4)
40–49	5·6 (5·4–5·9)	6·5 (6·4–6·7)	8·4 (8·1–8·8)	6·8 (6·7–6·9)	12·0 (11·8–12·3)	7·6 (7·5–7·6)
50–59	5·0 (4·7–5·3)	6·5 (6·3–6·7)	8·1 (7·7–8·4)	6·1 (6·0–6·3)	13·1 (12·8–13·5)	7·4 (7·3–7·5)
60–69	5·3 (4·9–5·8)	6·3 (6·1–6·5)	8·2 (7·7–8·6)	5·2 (5·1–5·4)	12·9 (12·5–13·3)	6·9 (6·8–7·1)
70+	5·3 (4·9–5·8)	6·2 (5·9–6·4)	9·2 (8·7–9·8)	4·4 (4·3–4·6)	13·5 (13·1–13·9)	6·6 (6·5–6·8)
Age, years (mean)	34·2 (34·0–34·5)	41·5 (41·3–41·7)	39·4 (39·1–39·6)	41·9 (41·8–42·0)	45·4 (45·2–45·6)	40·5 (40·4–40·6)
Race/Ethnicity						
White	3·2 (3·1–3·4)	2·7 (2·6–2·8)	6·2 (6·1–6·4)	5·7 (5·7–5·8)	10·7 (10·6–10·8)	6·4 (6·3–6·4)
Black	3·0 (2·8–3·2)	2·4 (2·3–2·5)	4·7 (4·3–5·0)	3·7 (3·6–3·8)	6·5 (6·2–6·9)	3·5 (3·4–3·5)
Asian	3·0 (2·0–3·9)	1·3 (1·0–1·6)	2·9 (2·1–3·6)	3·2 (2·9–3·6)	4·1 (3·3–5·0)	2·8 (2·6–3·0)
Mixed	5·4 (5·3–5·5)	6·2 (6·1–6·2)	7·7 (7·5–7·8)	4·5 (4·4–4·5)	4·4 (4·3–4·6)	5·5 (5·5–5·5)
Indigenous	15·0 (14·0–15·9)	2·3 (1·8–2·7)	25·1 (23·0–27·2)	3·6 (2·6–4·6)	10·6 (8·7–12·5)	11·6 (11·0–12·1)
Marital Status						
Single	6·3 (6·1–6·4)	5·4 (5·3–5·5)	8·7 (8·6–8·9)	6·0 (5·9–6·0)	10·2 (10·0–10·3)	6·6 (6·5–6·6)
Married/Civil union	6·3 (6·1–6·5)	5·8 (5·7–5·9)	7·6 (7·4–7·9)	4·9 (4·8–4·9)	10·2 (10·0–10·3)	6·2 (6·2–6·3)
Widow	3·8 (3·3–4·3)	4·4 (4·1–4·6)	6·7 (6·1–7·3)	3·8 (3·6–3·9)	10·0 (9·6–10·5)	5·1 (5·0–5·3)
Divorced/Separated	7·3 (6·5–8·0)	8·6 (8·2–9·0)	11·5 (10·8–12·2)	9·5 (9·2–9·7)	15·5 (14·9–16·0)	10·5 (10·3–10·6)
Schooling (%)						
None	6·8% (6·3–7·2)	9·5% (9·2–9·8)	4·2% (3·9–4·5)	1·8% (1·7–1·9)	2·3% (2·2–2·5)	4·3% (4·2–4·4)
1–3 years	13·7% (13·1–14·3)	18·9% (18·4–19·3)	11·4% (10·8–11·9)	8·6% (8·4–8·8)	12·4% (12·0–12·7)	12·5% (12·3–12·6)
4–7 years	27·6% (26·7–28·5)	22·9% (22·4–23·4)	23·7% (22·9–24·4)	21·8% (21·4–22·2)	25·6% (25·1–26·1)	23·5% (23·3–23·8)
8–11 years	31·8% (30·8–32·8)	19·5% (19·1–19·9)	28·5% (27·7–29·4)	28·7% (28·3–29·1)	27·2% (26·7–27·7)	26·4% (26·2–26·7)
12+ years	8·1% (7·6–8·6)	6·7% (6·4–6·9)	12·7% (12·1–13·2)	11·4% (11·1–11·6)	8·7% (8·4–9·0)	9·5% (9·4–9·7)
Missing values	11·7% (11·1–12·3)	22·3% (21·8–22·7)	19·3% (18·6–20·0)	27·5% (27·1–27·9)	23·5% (23·1–24·0)	23·5% (23·2–23·7)
Cause of death (%)						
Asphyxia/Hanging	82·3% (80·8–83·9)	72·1% (71·3–73·0)	69·9% (68·6–71·2)	65·4% (64·8–66·0)	74·2% (73·4–75·0)	70·7% (70·3–71·1)
Intoxication	6·8% (6·4–7·3)	13·4% (13·1–13·8)	12·0% (11·4–12·5)	11·1% (10·9–11·4)	8·3% (8·1–8·6)	10·8% (10·6–10·9)
Firearm	7·0% (6·5–7·5)	6·1% (5·9–6·4)	9·6% (9·1–10·1)	7·4% (7·2–7·6)	10·9% (10·6–11·3)	8·1% (8·0–8·2)
Other	3·6% (3·3–4·0)	8·1% (7·9–8·4)	8·4% (7·9–8·8)	15·9% (15·6–16·2)	6·3% (6·1–6·6)	10·3% (10·1–10·4)

Values are expressed as rates per 100,000 inhabitants (95% confidence intervals-CI). Rates were calculated by dividing the total number of deaths in each category by the corresponding person-years of exposure and multiplying by 100,000. Confidence intervals for rates were calculated assuming a Poisson distribution of deaths. Values for schooling and cause of death are presented as percentages (95% CI)-calculated using binomial distributions.

### Secondary objective 2: psychosocial care centers (CAPS)

Considering the importance of CAPS, Brazil's primary public mental health service, as a secondary research aim, we evaluated their distribution across cities and their association with suicide rates. We aggregated municipal suicide rates and the number of CAPS over three five-year periods: 2009–2013, 2014–2018, and 2019–2023, both for each of the five Brazilian regions and for Brazil as a whole. We processed the data to aggregate suicide rates and the number of CAPS over five-year periods. We merged the dataset with the geographic dataset of Brazilian cities to construct maps. We calculated Spearman's rank correlation between the number of CAPS and the ordinal categories of suicide rates (low, moderate, high) for each region. These analyses were ecological and descriptive.

### Ethics statement

Data collection occurred between November 2024 and June 2025. This study utilized deidentified publicly available secondary data; therefore, approval from a Research Ethics Committee was not required.

### Role of funding source

None of the funders of this work had any role in the study design, data collection, data analysis and interpretation, or in the report of the results and decision to submit.

## Results

### Profile of individuals who died by suicide in Brazil from 2009 to 2023

To characterize the sociodemographic profile of suicide deaths across Brazil's five geographical macroregions we used individual suicide death records from 2009 to 2023 obtained from SIM and IBGE population estimates to calculate suicide rates per 100,000 inhabitants. Of the 96 SIM variables, nine were related to suicide deaths: age, sex, date of death, race/ethnicity, schooling, place of death, municipality, state, and ICD-10 cause of death. Missing values for age and date of death were <1% and were excluded from the analyses. Missing values for race/ethnicity (2·2%), marital status (7·6%), and schooling (23·5%) were recorded as “ignored” in the SIM system when death certificates were incompletely filled and were not included in the data normalization process.

Over the 15-year study period, Brazil experienced approximately 174,038 suicide deaths, distributed across the regions: North, 12,706; Northeast, 40,366; Central-West, 16,179; Southeast, 64,319; and South, 40,468. Brazil showed a cumulative suicide rate of 5·7 deaths per 100,000 inhabitants, with substantial regional variation, ranging from 4·8 in the Northeast to 9·2 in the South. Furthermore, within each region, the cumulative suicide rates across cities showed substantial variability. Supplementary Table S2 presents the minimum and maximum suicide rates, as well as the mean, median, and standard deviation for each region.

**Fig. 2:**
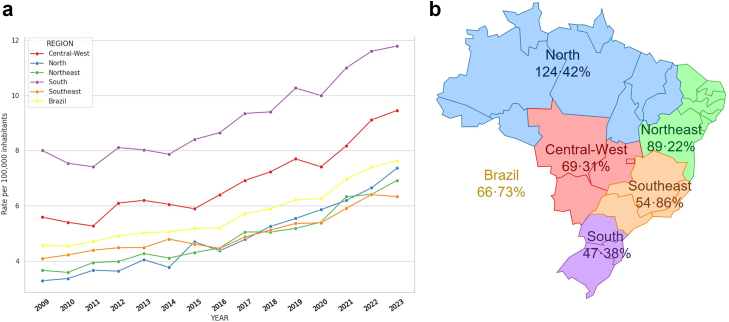
**Suicide rates by year in the five Brazilian regions. (a)** Suicide death rates per 100,000 inhabitants in Brazil (yellow) and their distribution across the five regions (North in red, Northeast in blue, Central-West in green, Southeast in orange, and South in purple). Rates were calculated for each year from 2009 to 2023. **(b)** Map of Brazil highlighting the five regions. Percentages represent the increase in suicide rates per 100,000 inhabitants from January 2009 to December 2023.

Suicide rates increased over time in all Brazilian regions between 2009 and 2023. Within-region analyses showed increases of 124·4% in the North, 89·2% in the Northeast, 69·3% in the Central-West, 54·9% in the Southeast, and 47·4% in the South. Over the same period, the national suicide rate increased by 66·7% (Fig. 2 and Supplementary Table S3), indicating a substantial rise in suicide mortality over time in Brazil.

Considering the Brazilian population, males had suicide rates 3·6 times higher than females, a pattern that was consistent across regions. However, the Northeast and South exhibited male-to-female rate ratios that exceeded the national average. In Brazil overall, the largest number of suicide deaths occurred among individuals aged 40–59 years. This pattern differed across regions. In the South, a higher proportion of suicide deaths was observed among older individuals (≥70 years), whereas in the North and Central-West the largest proportion occurred among young adults (20–29 years). a pattern observed in the North, Central-West, and South regions (alongside White individuals). Individuals who were divorced or separated represented the largest group in terms of marital status. Education level was categorized by years of schooling, with the most common range being 4–11 years (middle to high school) for the national data and most regions. However, in the Northeast the most common range was 1–7 years of schooling. Asphyxia was the leading cause of death in Brazil across all regions. However, the second most common method varied by region: intoxication was the second leading cause in Northeast, Central-West, and Southeast, whereas firearms were the second most common method in the North and South regions (Table 1).

### Suicide rate classification and healthcare infrastructure indicators

To address the primary aim of the study, the machine learning analyses included municipality-year observations integrating suicide rates with healthcare infrastructure indicators. Suicide rates were calculated annually using SIM mortality counts and IBGE population estimates, while healthcare infrastructure indicators from CNES, including establishments, workforce categories, equipment, teams, and specialized services, were aggregated by municipality and year. In the CNES dataset, the absence of a descriptor (missing value) for a municipality was interpreted as the absence of that resource or service and was coded as zero during aggregation.

The datasets for each region reflected the number of cities classified into different suicide rate categories (low, moderate, and high). The distribution was as follows: North, n = 3131 (low 56%, moderate 23%, high 21%); Northeast, n = 12 815 (low 49%, moderate 30%, high 21%); Central-West, n = 3512 (low 42%, moderate 26%, high 32%); Southeast, n = 12,852 (low 46%, moderate 22%, high 32%); and South, n = 10,226 (low 29%, moderate 22%, high 49%).

Using the healthcare infrastructure available in each city, the models predicted city-level suicide rate categories with accuracies ranging from 0·72 to 0·79 (Table 2). The models relied on multiple components of healthcare infrastructure to classify SR categories. Several features were consistently informative across regions, such as the number of SUS-affiliated establishments and professionals, the availability of basic and multispecialty care, and the presence of CAPS.

**Table 2:** Healthcare infrastructure associated with suicide rates across regions in Brazil.

Region	Metrics	Top 10 features
		Physicians
		Psychosocial Center (CAPS)
		SUS professionals
	Accuracy = 0·72 ± 0·04 (0·69–0·75)	SUS establishments
North	Precision = 0·71 ± 0·04 (0·64–0·73)	Mental Health specialization
	Sensitivity = 0·72 ± 0·02 (0·67–0·74)	Transsexual care
	F1-score = 0·72 ± 0·03 (0·68–0·72)	Patient Referral Coordination
		Community Health Workers
		Orthopedic specialization
		Administrative Center
		Basic care
		Pregnancy care
		SUS professionals
	Accuracy = 0·78 ± 0·02 (0·76–0·79)	Psychosocial Center (CAPS)
Northeast	Precision = 0·77 ± 0·02 (0·76–0·78)	Physicians
	Sensitivity = 0·76 ± 0·02 (0·74–0·77)	Community Health Workers
	F1-score = 0·76 ± 0·02 (0·75–0·77)	Basic Health Unit
		Mental Health specialization
		SUS establishments
		Dental Health Team
		SUS establishments
		SUS professionals
		Dental Health Team
	Accuracy = 0·79 ± 0·06 (0·76–0·81)	Basic Health Unit
Central-West	Precision = 0·75 ± 0·06 (0·72–0·77)	Pregnancy care
	Sensitivity = 0·76 ± 0·07 (0·74–0·79)	Physicians
	F1-score = 0·75 ± 0·06 (0·73–0·78)	Nephrology care
		Dentists
		Multispecialty Clinic
		Oncology care
		SUS establishments
		SUS professionals
		Dentists
	Accuracy = 0·73 ± 0·02 (0·72–0·75)	Hearing Specialization
Southeast	Precision = 0·71 ± 0·02 (0·69–0·72)	Nurses
	Sensitivity = 0·70 ± 0·01 (0·69–0·71)	Advisory Committees
	F1-score = 0·70 ± 0·02 (0·68–0·71)	Physicians
		Cardiac Specialization
		Transsexual care
		Basic Health Unit
		Psychosocial care center (CAPS)
		SUS establishments
		Dentists
	Accuracy = 0·73 ± 0·02 (0·71–0·74)	Practice Office
South	Precision = 0·72 ± 0·02 (0·70–0·73)	Hearing Specialization
	Sensitivity = 0·73 ± 0·01 (0·72–0·74)	Diagnostic equipment
	F1-score = 0·72 ± 0·01 (0·71–0·73)	Pregnancy care
		Basic Health Unit
		Nurses
		Specialized Emergency Unit

The evaluation metrics [mean ± standard deviation (95% confidence interval)] represent the weighted average of the different classes results on the test set (20% of the total data set for each region on five folds). Healthcare infrastructure features represent the feature count (e.g.-number of nurses) and are the top features considered by the models to predict the suicide rate.

**Fig. 3:**
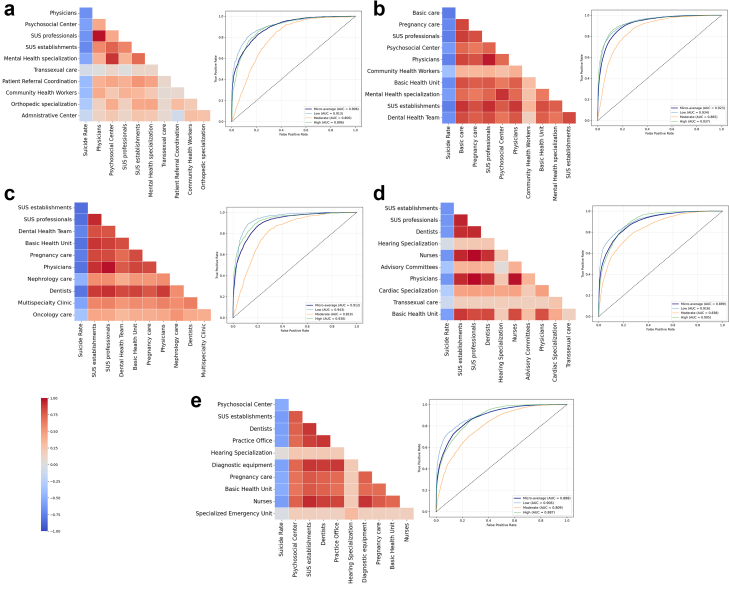
**Correlations between suicide rates and the key features considered by the models and AUROC (Area Under the Curve of the Receiver Operating Characteristic) of the predictions made by each model.** In the correlations, blue indicates a negative correlation, while red represents a positive correlation. In the AUROC, the y-axis shows the true suicide rate, and the x-axis shows the predicted suicide rate. The AUCROC values were computed considering the prediction of each class against the other two classes. **(a)** North, **(b)** Northeast, **(c)** Central-West, **(d)** Southeast, and **(e)** South regions.

Table 2 summarizes the evaluation metrics and highlights the top ten most influential variables for classifying suicide rates. Fig. 3 presents the AUROC, with the AUC values for considering the prediction of one class against the two others. Overall, the models performed best in classifying the low and high suicide rate categories, while accuracy was lower for the moderate category (also shown in Supplementary Figure S2). Notably, features included in the models were negatively correlated with suicide rates (Fig. 3), suggesting that higher values of these key variables are associated with lower suicide rates across Brazil's regions.

### Psychosocial care centers (CAPS)

**Fig. 4:**
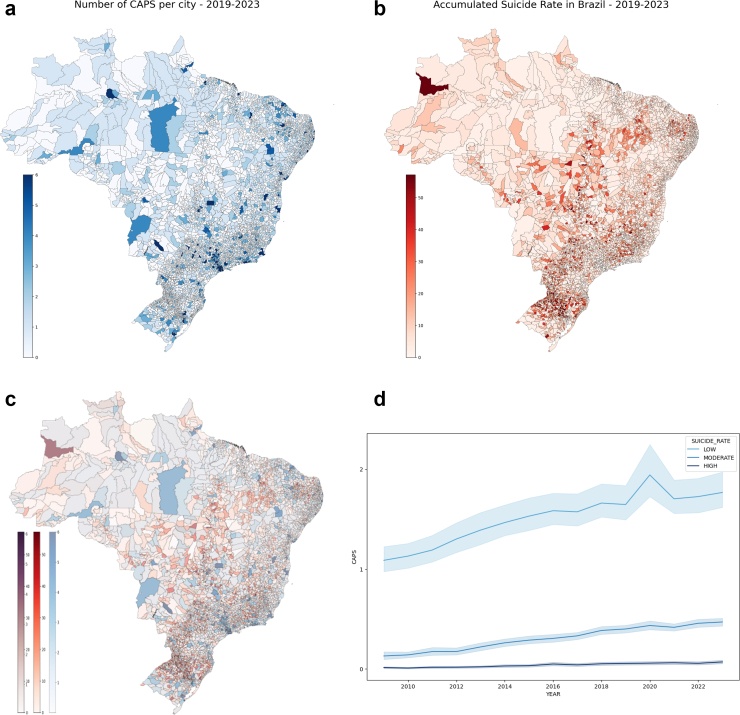
**Association between suicide rates and CAPS in Brazil.** Panels **(a)** and **(b)** show, respectively, the number of CAPS and the cumulative suicide rate for each city. Panel **(c)** presents a combined map overlaying both datasets. Darker colors indicate values in the 99th percentile. Panel **(d)** depicts the distribution of CAPS across cities per year grouped by low, moderate, and high suicide rate categories. CAPS, Psychosocial Care Centers.

As an additional secondary aim, we evaluated CAPS, which are central components of the country's community mental health network. Although CAPS coverage is relatively similar across regions (approximately 1·3 units per 100,000 inhabitants), suicide rates vary widely. Assessing CAPS at the city level, therefore, provides a more informative perspective. City-level mapping (Fig. 4) allows local differences in CAPS availability and suicide rate patterns to emerge, highlighting heterogeneity that would be obscured in regional averages.

Across the three periods evaluated (2009–2013, 2014 to 2018, and 2019 to 2023), the correlation between the number of CAPS and cumulative suicide rates were consistently negative and strengthened over time (p < 0·01): −0·30, −0·41, and −0·54, respectively, with a higher increase in the different regions (−0·20 to −0·81, Supplementary Table S4 and Supplementary Figures S3 and S4). Fig. 4 presents maps of Brazil showing the cumulative suicide rates alongside the number of CAPS per city for 2019–2023, with additional maps for other periods provided in Supplementary Figure S5. Spearman's rank correlation was used to assess population-level associations between CAPS availability and suicide-rate categories. These analyses are descriptive and do not imply causality.

## Discussion

This study combined national mortality and healthcare infrastructure data to assess regional patterns and related features associated with suicide rates in Brazil. First, we characterized the sociodemographic and geographic profile of individuals who died by suicide in Brazil. Second, we used healthcare infrastructure features to predict city-level suicide rate categories, identifying the components most informative for distinguishing areas with low, moderate, or high suicide rates. Finally, we evaluated the distribution of CAPS, public multidisciplinary units dedicated to mental health care, and their association with suicide rates across regions. Our analysis shows that general healthcare infrastructure was among the most informative features for classifying suicide rate categories (low, moderate, high) across regions. These features included basic care services, diagnostic services, general clinics, multispecialty primary care units, pregnancy care, and healthcare professionals such as physicians, dentists, and nurses. The number of SUS (Unified Health System) establishments and healthcare professionals also emerged as key features across regions. We further demonstrated an association between the number of CAPS per municipality and suicide rates.

In the descriptive analyses, the South region showed consistently higher recorded suicide mortality, while the North and Northeast exhibited the most pronounced increases over the study period. Suicide rates also varied widely within regions, ranging from very low values (close to zero) to very high rates (exceeding 80 deaths per 100,000 inhabitants; Supplementary Table S2 and Fig. 1). Descriptive patterns further indicated regional differences in demographic characteristics and methods: suicide deaths occurred at older ages in the South, Indigenous populations appeared to be disproportionately represented among deaths, and firearms and intoxication were the second and third most frequently reported methods, both of which may be responsive to regulatory measures.^27^ These observations highlight regional heterogeneity in suicide mortality and associated characteristics. These comparisons should be interpreted cautiously, as regional differences in population composition (e.g., age structure and demographic distribution) may influence crude rate estimates. Given the ecological design, these patterns do not imply causality but may help inform region-specific public health planning.

City infrastructure, particularly the quality and accessibility of healthcare, appears to be associated with suicide rates.^10^^,^^28^^,^^29^ In this study, we evaluated the influence of healthcare infrastructure on suicide rates across Brazil's five regions using a machine learning model, which demonstrated good predictive performance, with accuracy ranging from 72% to 79%. The features considered by the model varied in order of importance, but general healthcare infrastructure, encompassing basic care services, diagnostic services, general clinics, multispecialty primary care units, pregnancy care, and healthcare professionals such as physicians, dentists, and nurses, emerged as the most significant factors. Many of these professionals operate within primary care units, providing basic and multispecialty services.

Although direct cross-country comparisons are challenging, the available literature demonstrates that health-system and social-infrastructure indicators are indeed associated with suicide rates. Pirkis et al.^8^ emphasize that suicide is driven by multiple structural determinants, including access to health services, but also note substantial variability in data quality across countries, which limits straightforward international comparisons. Nevertheless, ecological studies consistently show that greater availability of social and health-related infrastructure is linked to lower suicide rates. For example, Zhang et al.,^30^ report that U.S. counties with stronger social and service infrastructure have lower suicide mortality, while studies from Slovenia^31^ and the United States^32^ demonstrate that regions with higher densities of psychiatrists or without mental-health provider shortages have lower suicide rates. Similarly, recent analyses in the European Union indicate that accessibility of healthcare, reflected in the number of psychiatrists, availability of medical care, and lower unmet health needs, explains a substantial portion of suicide-rate variance across countries.^33^ Together, these findings show that international comparisons based specifically on health infrastructure remain limited, especially within LMICs. Still, there is consistent evidence that broader health-service availability and accessibility are relevant correlates of suicide, supporting our focus on infrastructure-related determinants.

The number of SUS (Unified Health System) establishments and healthcare professionals were also consistent among the most influential variables across regions. Brazil's healthcare system is decentralized, prioritizing primary care and outpatient specialized services over hospitalization, and more than 70% of the population relies exclusively on public services. Service distribution is guided by demographic density and economic indicators: smaller cities typically provide only primary care, whereas larger cities offer more comprehensive services and act as regional hubs delivering healthcare to surrounding areas.^34^ Our findings support an association between SUS infrastructure and suicide rates. Expanding access to clinicians and nurses in cities with high suicide rates could impact mortality, while the models also highlighted the contribution of features such as dental professionals and community workers, suggesting that general well-being is also associated with suicide risk. Other services, such as pregnancy care and diagnostic facilities, are also relevant. Pregnancy and the postpartum period are high-risk periods for mental health conditions,^35^, ^36^, ^37^ and prenatal care in Brazil often includes mental health support, which may help explain the significance of pregnancy care in suicide risk. Although healthcare infrastructure plays an important role, it represents only one component of the broader environment influencing suicide risk. Additional contextual factors, such as housing quality, education, access to leisure and social resources, and food and water availability, may contribute to either increasing or reducing suicide rates.

Considering the importance of mental health care, in a separate aim of our work, we examined CAPS units more closely, focusing on their geographical distribution (municipal-level) and their association with suicide rate categories. CAPS is a type of healthcare facility focused on treating psychiatric patients. CAPS are divided into seven classes: CAPS I serves people of all ages experiencing psychological distress and requires a minimum population of 15,000 inhabitants. CAPS II targets adults with more severe psychological distress; CAPSi, serves children and adolescents. CAPS AD (Alcohol and Drugs), addresses substance abuse-related psychological suffering across all ages. These last three require municipalities with at least 70,000 inhabitants. Higher-intensity services—CAPS III, CAPS AD III, and CAPS AD IV—cater to all age groups with severe psychiatric conditions or substance abuse, operate 24 h, and include hospital beds. CAPS III and CAPS AD III serve populations of at least 150,000 inhabitants, while CAPS AD IV requires 500,000 inhabitants.^38^

Previous reports from Southern Brazil support the hypothesis that the implementation of CAPS is negatively correlated with psychiatric hospitalization.^39^ However, the present study is the first to systematically examine the association between CAPS coverage and suicide rates across the country. A higher concentration of CAPS tends to display a lower suicide rate, independent of the Brazilian region (Supplementary Figure S3). Brazil has 3019 CAPS, distributed across 2194 of the 5570 different cities. Although the growing rate of CAPS in the different regions, it is not enough to compensate the growing suicide rate. By 100,000 inhabitants, the regions have similar number of CAPS: North—0·9; Northeast—1·7; Central-West—1·0; Southeast—1·1; South—1·5; Brazil—1·3. As shown, in Supplementary Figure S4, the presence of even one CAPS is associated with moderate and low suicide rates. CAPS teams integrate nurses, psychiatrists, therapeutic assistants and more, and are “open doors,” offering community mental health services to everyone that needs it.

Furthermore, considering the increasing number of suicides among young people, CAPSi may be relevant for regions with rising youth suicide rates, because it has professionals specialized in children and adolescent treatment, although causal effects cannot be inferred from ecological data. Suicide rates among children and adolescents in the North, Central-West, and South regions of Brazil are higher than the national average, reaching approximately five deaths per 100,000 inhabitants. Therefore, it is also crucial to implement targeted mental health awareness and suicide prevention programs in these areas. School-based preventive interventions for children and adolescents are cost-effective and have been shown to reduce future suicide attempts and deaths.^40^

Similarly, CAPS AD is focused on treating people suffering with substance abuse, particularly alcohol and crack, which are major risk factors for suicide.^1^ Suicide risk is also higher in inpatients, and as demonstrated in three large capitals of Brazil (São Paulo, Rio de Janeiro, and Porto Alegre), the presence and increase of CAPS is correlated with the decrease of psychiatric hospitalizations.^39^^,^^41^ These findings highlight the potential of CAPS to serve not only as treatment centers but also as proactive elements in national suicide prevention strategies.

Suicide is a complex phenomenon that requires investigation from multiple perspectives. In this study, we leveraged data from the Brazilian Unified Health System to characterize individuals who died by suicide and to examine how healthcare infrastructure relates to suicide rates across the country's regions. While clinical data can help predict suicide risk at the individual level, broader population-based analyses can inform interventions that modify systemic factors, potentially benefiting society at large.^17^ However, several factors should be considered when interpreting these findings. Comparisons across regions and demographic groups must be made cautiously, as suicide rates were calculated using population denominators that vary over time and across age, sex, and regional distributions. Demographic shifts such as population aging, migration, and differential regional growth may influence rate estimates independently of true changes in suicide risk. In addition, suicide mortality is shaped by multiple contextual determinants beyond healthcare infrastructure, including economic instability, unemployment, social inequality, urbanization, access to lethal means, and substance use trends. Because our analyses were ecological and based on machine learning models, the results should be interpreted as correlational rather than causal. Data-related limitations should also be acknowledged, including incomplete or inconsistently reported SIM and CNES records, data anonymization that removed potentially informative variables, and the inability to distinguish between healthcare professionals' specialties. Additionally, findings at the aggregate level cannot be assumed to be consistent at the individual level due to the ecological fallacy. Furthermore, improvements in national health information systems, particularly expanded coverage and enhanced classification of external causes in the Mortality Information System (SIM), may have increased the identification of suicide deaths over time, meaning that part of the observed increase may therefore reflect improved ascertainment rather than a true rise in incidence. Despite these limitations, our findings provide relevant insights into the relationship between healthcare infrastructure and suicide mortality and may help inform targeted public health strategies and prevention efforts across Brazil.

### Conclusion and public health implications

Suicidal behavior is a multifaceted phenomenon. This study shows that indicators of healthcare infrastructure, including general services, specialized care, and the distribution of Psychosocial Care Centers (CAPS), are closely associated with regional patterns of suicide rates in Brazil. General healthcare services, workforce availability, and SUS establishments emerged as features that help differentiate suicide rate categories across the country. Regional differences in suicide prevalence, demographic profiles, and methods reinforce the need for context-sensitive and region-specific approaches. Our findings highlight the relevance of strengthening and expanding primary care and accessible mental health services, alongside culturally and age-appropriate strategies and school- or community-based initiatives, as part of broader public health planning. Future studies could build on this approach by incorporating additional city-level indicators, such as urban-rural distribution, industrialization, employment, education, and sanitation, to better understand how structural characteristics align with suicide rate patterns. Reducing suicide remains a major public health challenge, particularly in LMICs, and identifying the structural factors most consistently associated with suicide outcomes can support the development of more targeted and locally responsive policies.

## Contributors

CAP, MGS, JTC, and MPK conceived and designed the study. CAP was responsible for data selection, integration, and analysis. JTC, JMN, and MPK supervised the data analysis and discussion. CAP, JMN, and MPK drafted the manuscript. All authors read and approved the final manuscript. CAP and MPK contributed to manuscript revision and submission.

## Data sharing statement

The codes for the methods and results are deposited in the following Github repositories and will be available upon publication: https://github.com/caibealves/Suicide_Rate, https://github.com/caibealves/BrazilianHealthcareDatasets/.

## Disclosure of AI or AI-assisted technologies use

We used ChatGPT 5·0 assistance to correct the language and punctuation of the text.

## Editor note

The Lancet Group takes a neutral position with respect to territorial claims in published maps and institutional affiliations.

## Declaration of interests

All authors declare no competing interests.
