# Supplementary material for: Analysis of health infrastructure and suicide rates in Brazil: a nationwide ecological spatial–temporal study, 2009–2023
Source: Lancet Reg Health Am. 2026 Jun 3;60:101519. doi: 10.1016/j.lana.2026.101519 (PMC13254669; doi:10.1016/j.lana.2026.101519)
Supplement: Supplemental Material [file mmc1.pdf]

Analysis of Health Infrastructure and Suicide Rates in Brazil: a nationwide ecological spatial-temporal study, 2009–2023

List of contents:

Supplementary Table 1. Healthcare infrastructure descriptors

Supplementary Table 2. Municipal variability of suicide rates within each region

Supplementary Figure 1. Violin plot showing the accumulated distribution of suicide rate in each region from 2009 to 2023

Supplementary Table 3. Normalized rate per 100 000 inhabitants from all Brazilian regions per year

Supplementary Figure 2. Confusion matrices summarizing model predictions obtained through nested cross-validation.

Supplementary Table 4. Correlation between Suicide Rates and CAPS number per region

Supplementary Figure 3. Suicide rate category by year considering the number of CAPS.

Supplementary Figure 4. Association between the suicide rate categories and number of CAPS corresponding to 5-year periods in Brazil.

Supplementary Figure 5. Brazilian maps showing the accumulated suicide rate or number of CAPS corresponding to 5-year periods.

Supplementary Figures

Supplementary Table 1. Healthcare infrastructure descriptors

Team qualifications	Teams	Equipment	Specialized services	Professionals	Types of units
Teaching	Family health	Image diagnosis	Prison	Biomedical scientist	Primary healthcare
Phylantropy	Oral health	Infrastructure	Adolescent health	Speech therapist	Multispecialty primary care
Surgery	Health agents	Optical methods	Basic health	Biologist	Multispecialty office
Obesity	Prison health	Graphic methods	Cardiovascular	Physical educator	General hospital
Odontology	Indigenous health	Life maintenance	Committees	Radiologist	Mixed emergency
Ophthalmology	Primary care	Other	Diagnosis	Pegadogy	General emergency room
Mental Health	Mental health	Dentistry	Endocrinology	Optician	Specialized emergency room
Cardiovascular		Audiology	Health surveillance	Radiology technician	Medical office
Chronic diseases		Telehealth	Hearing health	Social worker	Mobile unit (river)
HIV/AIDS			Hemotherapy	Nurse	Specialist unit
Oncology			ICU	Pharmacist	Diagnostic center
Geriatric			Immunization	Physical/Occupational therapist	Mobile unit
Pregnancy			Indigenous health	Physician	Mobile urgency care
Nephrology			Mental Health	Nutricionist	Pharmacy
Physical rehabilitation			Nephrology	Dentist	Healthcare surveillance
Nutrition/ Gastrology			Neurology	Psychologist	Health insurance
Transplant			Nutrition/ Gastrology	Veterinarians	Childbirth center
Orthopedic			Obesity		Shortstay hospital
ICU			Oncology		Healthcare regulator
Urgency			Ophthalmology		Central laboratory
Administration			Oral health		Health department
Intermediary care			Orthopedic		Hemotherapy center
Transexual change			Pharmacy		Psychosocial care center
Trauma			Physical therapy		Family center
Genetic			Pneumology		Indigenous center
Sexual violence			Pregnancy		Educational center

Especialist			Rare diseases		Telehealth
Auricular			Rehabilitation		Urgency care regulator
Worker care			Reproductive health		Home care
			Sexual violence		Public health laboratory
			Smoking		Access center
			STI/HIV/AIDS		Notification center
			Surgery		Prevention center
			Teaching		Supply center
			Telehealth		Immunization center
			Transexual care		
			Transplant		
			Tuberculosis/ Hansens diseases		
			Urgency		

Supplementary Table 2. Municipal variability of suicide rates within each region

Region	Minimum	Maximum	Median	Mean	Standard Deviation
**Central-West**	0·48	87·92	10·53	14·68	12·89
**North**	0·25	91·07	6·04	10·16	11·33
**Northeast**	0·22	80·84	6·78	9·50	8·29
**South**	0·10	81·51	12·25	15·94	13·80
**Southeast**	0·03	123·53	7·70	11·36	11·65

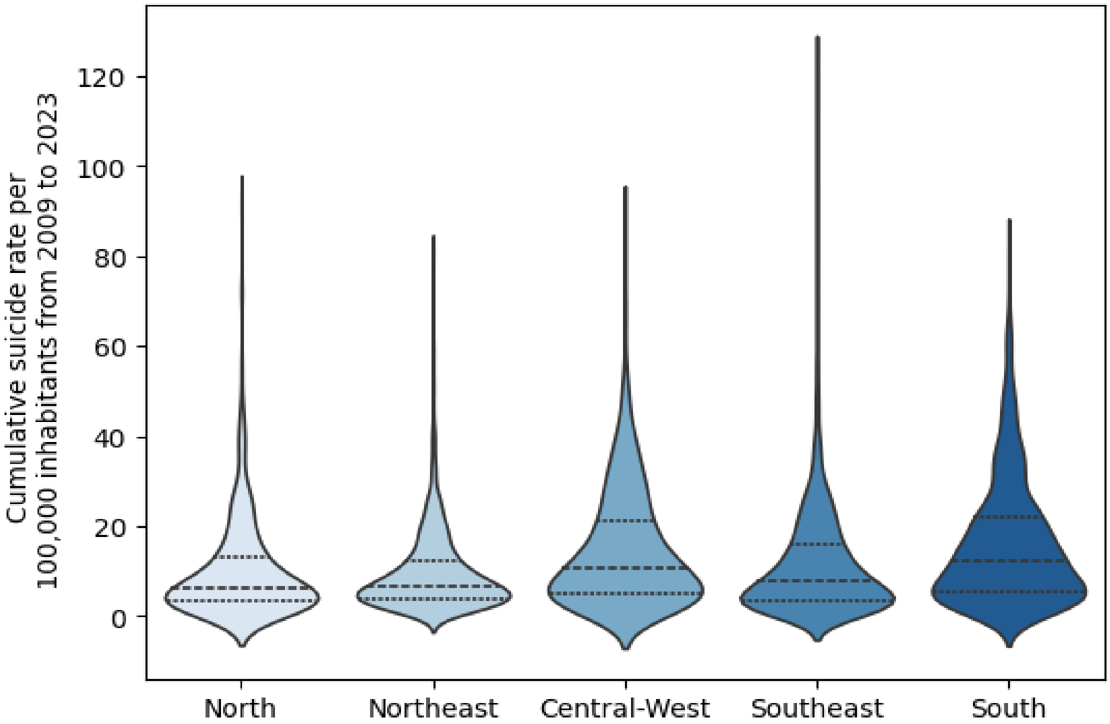

Supplementary Figure 1. Violin plot showing the accumulated distribution of suicide rate in each region from 2009 to 2023

Supplementary Table 3. Normalized rate per 100 000 inhabitants from all Brazilian regions per year

REGION	YEAR	Normalized Rate	CI lower	CI upper
	2009	5·6	5·2	6·0
	2010	5·4	5·0	5·8
	2011	5·3	4·9	5·6
	2012	6·1	5·7	6·5
	2013	6·2	5·8	6·6
	2014	6·1	5·7	6·4
	2015	5·9	5·5	6·3
**Central-West**	2016	6·4	6·0	6·8
	2017	6·9	6·5	7·3
	2018	7·2	6·8	7·6
	2019	7·7	7·3	8·1
	2020	7·4	7·0	7·8
	2021	8·2	7·7	8·6
	2022	9·1	8·7	9·6
	2023	9·5	9·0	9·9
	2009	3·3	3·0	3·6
	2010	3·4	3·1	3·6
	2011	3·7	3·4	4·0
	2012	3·6	3·3	3·9
	2013	4·0	3·7	4·3
**North**	2014	3·8	3·5	4·1

	2015	4·7	4·4	5·0
	2016	4·4	4·1	4·7
	2017	4·8	4·5	5·1
	2018	5·3	4·9	5·6
	2019	5·5	5·2	5·9
	2020	5·9	5·5	6·2
	2021	6·2	5·8	6·6
	2022	6·7	6·3	7·0
	2023	7·4	7·0	7·8
	2009	3·7	3·5	3·8
	2010	3·6	3·4	3·7
	2011	3·9	3·8	4·1
	2012	4·0	3·8	4·1
	2013	4·3	4·1	4·4
	2014	4·1	3·9	4·3
	2015	4·3	4·1	4·5
**Northeast**	2016	4·5	4·3	4·6
	2017	5·1	4·9	5·2
	2018	5·1	4·9	5·2
	2019	5·2	5·0	5·4
	2020	5·4	5·2	5·6
	2021	6·3	6·1	6·5
	2022	6·4	6·2	6·6
	2023	6·9	6·7	7·1
	2009	8·0	7·7	8·3
	2010	7·5	7·2	7·9
	2011	7·4	7·1	7·7
	2012	8·1	7·8	8·4
	2013	8·0	7·7	8·4
	2014	7·9	7·5	8·2
	2015	8·4	8·1	8·7
**South**	2016	8·7	8·3	9·0
	2017	9·3	9·0	9·7
	2018	9·4	9·1	9·8
	2019	10·3	9·9	10·6
	2020	10·0	9·6	10·4
	2021	11·0	10·6	11·4
	2022	11·6	11·2	12·0
	2023	11·8	11·4	12·2
	2009	4·1	4·0	4·2
	2010	4·2	4·1	4·4
	2011	4·4	4·2	4·5
	2012	4·5	4·3	4·6
	2013	4·5	4·3	4·6
	2014	4·8	4·6	4·9
	2015	4·6	4·5	4·8
	2016	4·5	4·3	4·6
**Southeast**	2017	4·9	4·7	5·0

	2018	5·1	5·0	5·3
	2019	5·4	5·2	5·5
	2020	5·4	5·2	5·5
	2021	5·9	5·7	6·1
	2022	6·4	6·2	6·6
	2023	6·3	6·2	6·5
	2009	4·6	4·5	4·7
	2010	4·5	4·4	4·6
	2011	4·7	4·6	4·8
	2012	4·9	4·8	5·0
	2013	5·0	4·9	5·1
	2014	5·1	5·0	5·2
	2015	5·2	5·1	5·3
**Brazil**	2016	5·2	5·1	5·3
	2017	5·7	5·6	5·8
	2018	5·9	5·8	6·0
	2019	6·2	6·1	6·3
	2020	6·3	6·2	6·4
	2021	7·0	6·9	7·1
	2022	7·4	7·3	7·5
	2023	7·6	7·5	7·7

CI, 95% confidence interval for suicide rates were calculated assuming a Poisson distribution of deaths. The standard error of the rate was estimated as the square root of the number of deaths divided by the corresponding person-years of exposure, scaled to rates per 100 000 inhabitants.

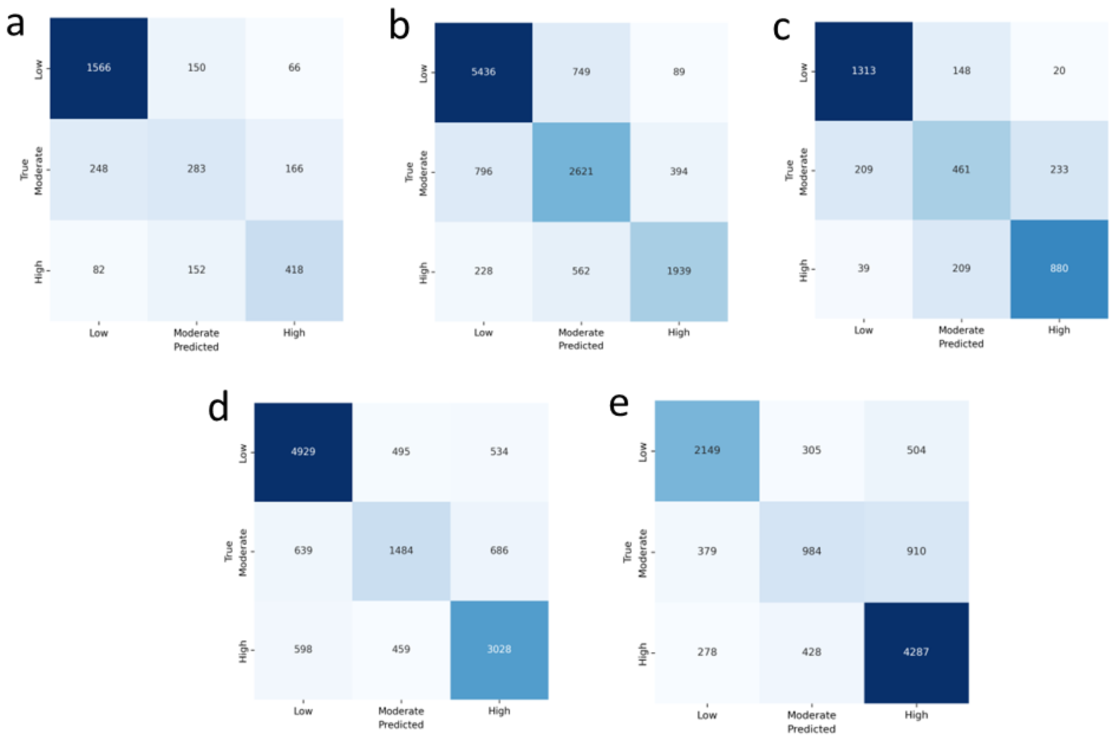

Supplementary Figure 2. Confusion matrices summarizing model predictions obtained through nested cross-validation. Rows represent the true suicide-risk classes, and columns represent the predicted classes. Color intensity indicates the number of municipalities in each cell (a) North, (b) Northeast, (c) Central-West, (d) Southeast, and (e) South regions.

Supplementary Table 4. Correlation between Suicide Rates and CAPS number per region

Region	2009-2013	2014-2018	2019-2023
**North**	ρ = -0·36<br>R^2 = 0·01	ρ = -0·60<br>R^2 = 0·09	ρ = -0·71<br>R^2 = 0·27
**Northeast**	ρ =-0·47<br>R^2 = 0·09	ρ = -0·63<br>R^2 = 0·22	ρ = -0·72<br>R^2 = 0·35
**Central-West**	ρ = -0·51<br>R^2 = 0·07	ρ = -0·67<br>R^2 = 0·21	ρ = -0·73<br>R^2 = 0·28
**Southeast**	ρ = -0·24<br>R^2 = 0·00	ρ = -0·58<br>R^2 = 0·11	ρ = -0·81<br>R^2 = 0·36
**South**	ρ = -0·20<br>R^2 = 0·00	ρ = -0·54<br>R^2 = 0·06	ρ = -0·79<br>R^2 = 0·32
**Brazil**	ρ = -020<br>R^2 = 0·09	ρ = -0·41<br>R^2 = 0·14	ρ = -0·54<br>R^2 = 0·20

Spearman's rank correlation of the accumulated suicide rate and number of CAPS corresponding to 5-year periods computed in the cities of Brazilian regions. For all the correlations, p < 0·01. CAPS, Psychosocial Care Center.

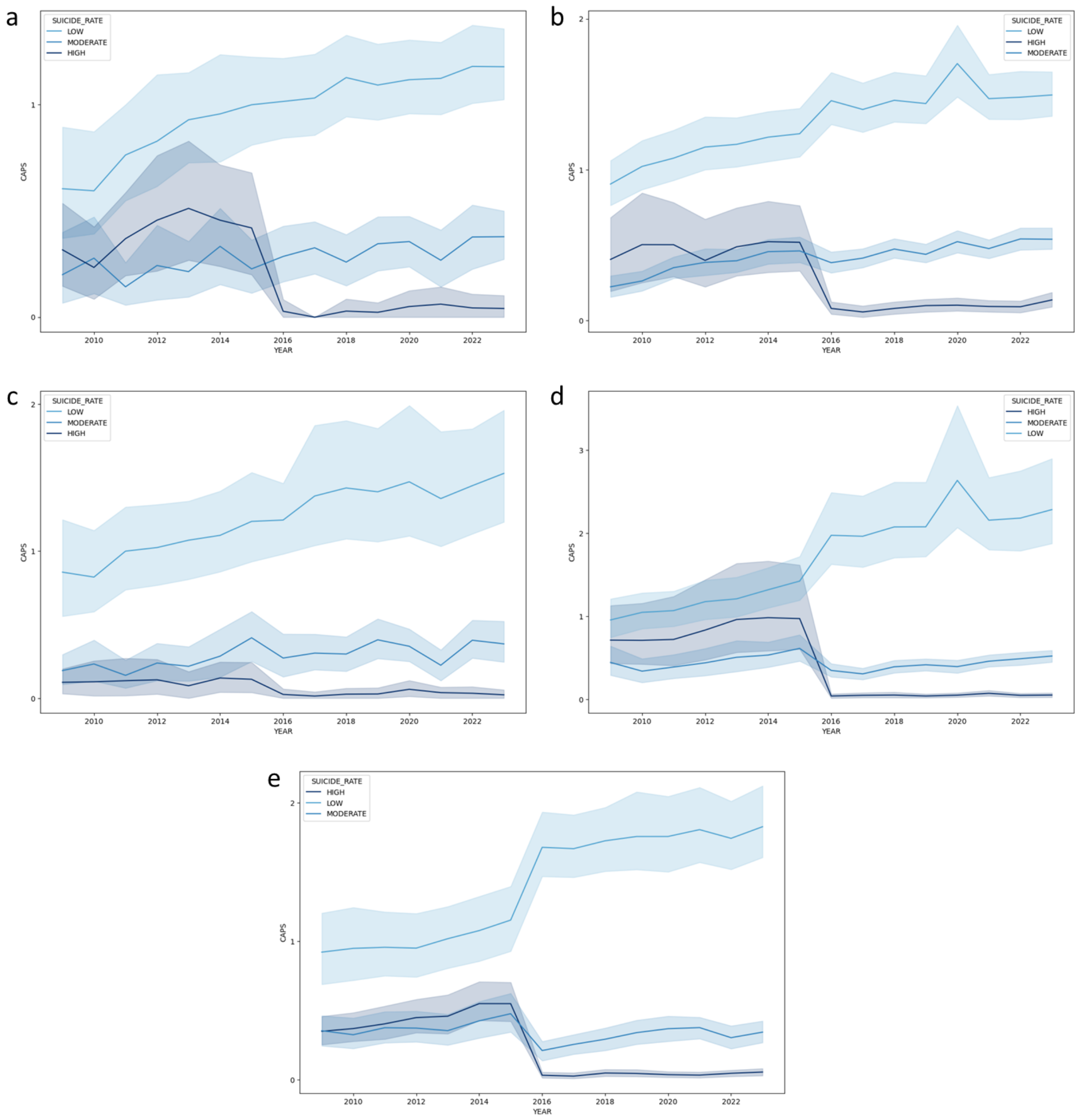

Supplementary Figure 3. Suicide rate category by year considering the number of CAPS. (a) North, (b) Northeast, (c) Central-West, (d) Southeast, and (e) South regions· CAPS, Psychosocial Care Centers.

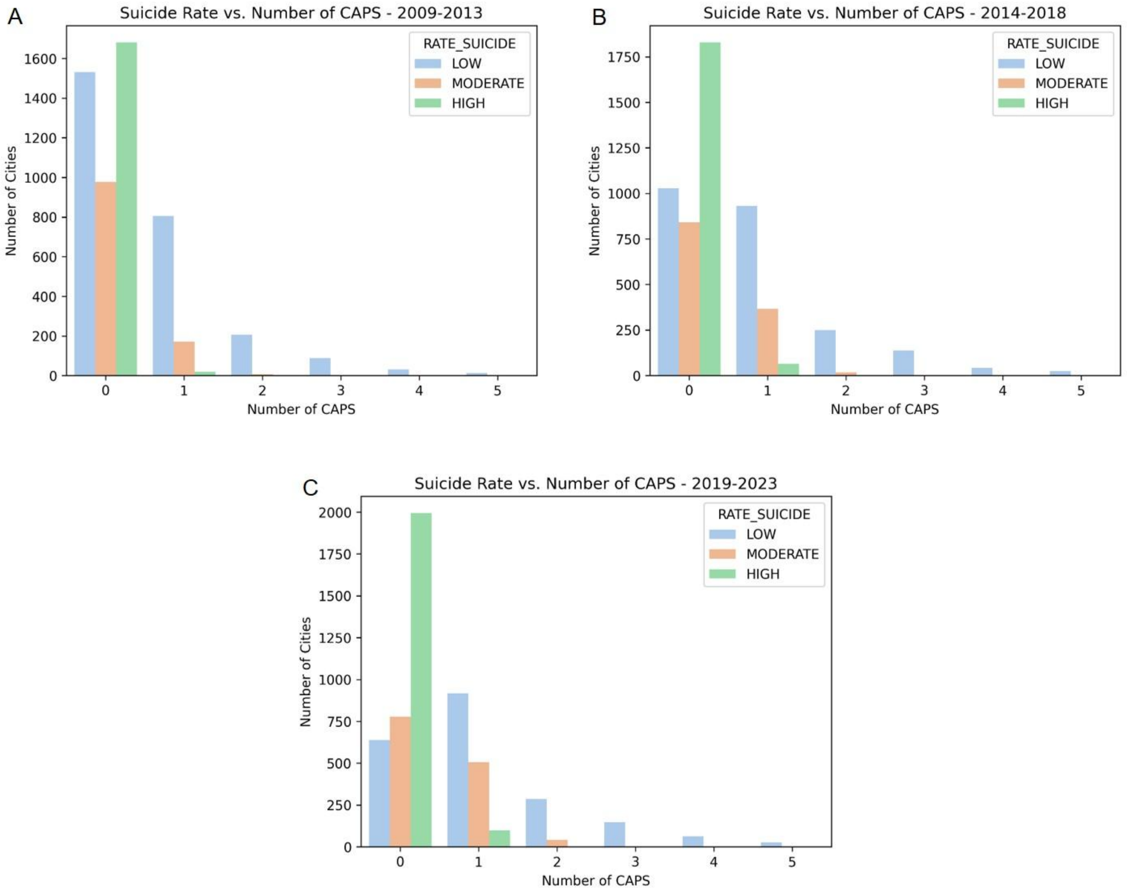

Supplementary Figure 4. Association between the suicide rate categories and number of CAPS corresponding to 5-year periods in Brazil. CAPS, psychosocial care centers.

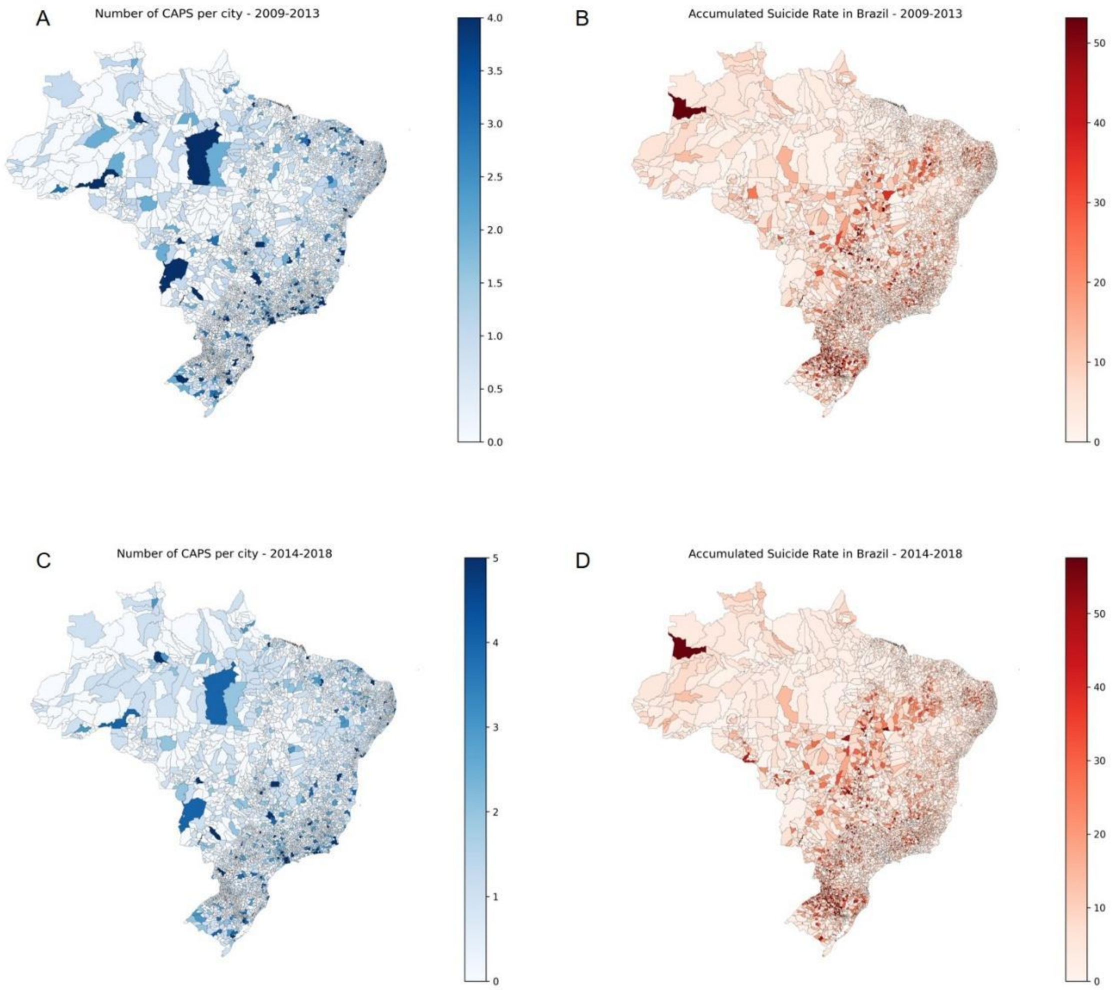

Supplementary Figure 5. Brazilian maps showing the accumulated suicide rate or number of CAPS corresponding to 5-year periods. The stronger colors represent the 99th percentile. CAPS, psychosocial care centers.
